# Electrolyte Additives as Pathway Selectors in Early SEI Formation

**DOI:** 10.1021/acs.jpclett.6c00492

**Published:** 2026-05-07

**Authors:** Fernando A. Soto

**Affiliations:** School of Science, Engineering, and Technology, Penn State Harrisburg, Middletown, Pennsylvania 17057, United States

## Abstract

Electrolyte additives such as fluoroethylene carbonate (FEC) and vinylene carbonate (VC) improve lithium-ion battery performance, yet whether they shift product distributions along common pathways or select fundamentally different decomposition channels remains debated. Here, ensemble *ab initio* molecular dynamics simulations coupled with machine learning structural analysis quantify how electrolyte composition controls access to configuration space during early solid-electrolyte interphase (SEI) formation. Analysis of 15 independent trajectories across baseline, FEC-rich, and VC-rich formulations using SOAP descriptors, UMAP, and HDBSCAN reveals that each composition occupies nonoverlapping structural regions characterized by distinct Li–O coordination environments and composition-dependent Li–F signatures. Diagnostic comparison of initial and evolved configurations, including Li-centered SOAP descriptors that isolate emergent structural changes from compositional encoding, and supported by time-resolved SOAP analysis, provides evidence that this separation reflects emergent structural divergence during decomposition. These results suggest that electrolyte additives function as pathway selectors during early interfacial reaction, offering a framework for rational electrolyte design.

## Graphical abstract

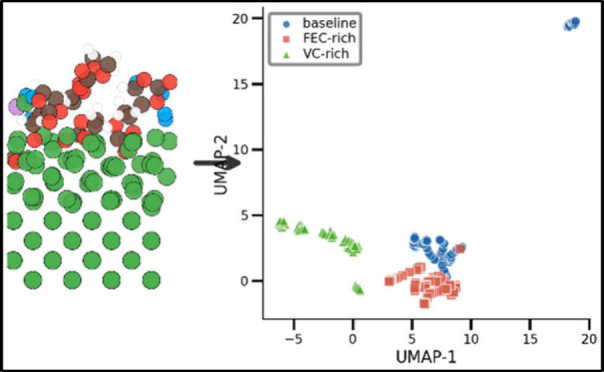

The solid-electrolyte interphase (SEI) governs lithium-ion battery performance by mediating ion transport while blocking electron transfer, yet rational design of SEI-forming electrolytes remains challenging because the relationship between molecular composition and interfacial structure is incompletely understood.^1–3^ Electrolyte additives such as fluoroethylene carbonate (FEC) and vinylene carbonate (VC) demonstrably improve cycling stability and Coulombic efficiency,^4–6^ and recent mechanistic studies have begun to reveal how these additives modify decomposition chemistry. Notably, Intan and Pfaendtner^7^ demonstrated using density functional theory (DFT) and *ab initio* molecular dynamics (AIMD) simulations that ethylene carbonate (EC) preferentially decomposes through an S2 pathway (nucleophilic attack on ethylene carbon) that promotes alkoxide regeneration and continuous oligomerization, whereas FEC favors the S1 pathway (attack on carbonyl carbon) that does not regenerate alkoxides and produces a self-terminating SEI. Similarly, Hou et al.^8^ showed that Li^+^-coordinated FEC reduces approximately 0.3 V higher than EC, intercepting electrons before EC can react and thereby altering the onset and sequence of electrolyte decomposition.

These mechanistic insights suggest that additives do not merely shift thermodynamic product distributions but rather select between fundamentally different reaction channels. If FEC and VC redirect decomposition into distinct pathways, then the structural organization of the resulting SEI should reflect this pathway divergence from the earliest stages of formation. Indeed, cryo-electron microscopy studies have revealed that different electrolyte compositions produce qualitatively different SEI architectures, including heterogeneous mosaic structures with dispersed nanocrystalline domains and more uniform, multilayer interphases, that correlate with battery performance.^9–11^ Han et al.,^12^ using cryo-TEM of Na metal electrodes, demonstrated that different additives produce distinct structural architectures, with FEC generating a multilayer SEI comprising an NaF-rich amorphous phase, suggesting that similar additive-dependent structural diversity may operate in Li systems.

AIMD simulations have provided essential molecular-level insight into early SEI formation, revealing solvent ring-opening mechanisms,^13–15^ radical intermediates,^16,17^ and composition-dependent reduction sequences.^18,19^ However, computational studies typically rely on one or a small number of trajectories, limiting the ability to assess whether observed structural features are representative or trajectory-specific. This limitation is particularly relevant for understanding pathway selection, where the question is not simply what structures form but which regions of configuration space different compositions can access. Wang et al.^20^ noted that the liquid electrolyte’s complexity means reactions depend strongly on local chemical environment, making statistical sampling essential. Recent studies have begun to address this gap: Zhang et al.^21^ combined molecular dynamics simulations and experimental studies for water-in-salt electrolytes, and kinetic Monte Carlo approaches have been used to study electrolyte solution and SEI formation.^22,23^

Here, this study employs an ensemble AIMD approach coupled with machine learning structural analysis to examine whether different electrolyte compositions occupy distinct regions of structural phase space during early interfacial reactions that lead to SEI formation. The work uses smooth overlap of atomic positions (SOAP) descriptors^24^ to encode local atomic environments, uniform manifold approximation and projection (UMAP)^25^ for dimensionality reduction, and hierarchical density-based spatial clustering (HDBSCAN)^26^ to identify structural motifs. This pipeline has proven effective for analyzing structural motifs in large-scale material data sets and evolving metastable states in related systems.^27,28^ Applied to 15 independent simulations spanning baseline, FEC-rich, and VC-rich electrolytes on Li(100), this approach reveals partitioning of configuration space by composition, providing evidence that additives may function as pathway selectors from the earliest stages of interfacial reaction.

## Computational Methods

All AIMD simulations were performed using VASP 6.4 ^29–32^ with the Perdew–Burke–Ernzerhof (PBE) exchange–correlation functional^33^ and Grimme D3­(BJ) dispersion correction,^34^ a 520 eV plane-wave cutoff, Γ-point sampling, and projector augmented-wave (PAW) pseudopotentials.^35^ Three electrolyte compositions were studied on a four-layer Li(100) slab (3.50 Å lattice constant, bottom two layers fixed): (1) baseline: 3 EC, 2 FEC, 2 VC, and 1 LiPF_6_; (2) FEC-rich: 3 EC, 4 FEC, and 1 LiPF_6_; (3) VC-rich: 3 EC, 4 VC, and 1 LiPF_6_. Five independent initial configurations were generated for each composition using randomized molecular placements and randomized initial velocities, yielding 15 total simulations. Trajectories were propagated for approximately 5 ps at 300 K using a Nosé–Hoover thermostat^36^ with a 1 fs time step.

The simulation model represents a qualitative interfacial configuration capturing the earliest stages of electrolyte-electrode contact, rather than a bulk electrolyte environment. The small number of electrolyte molecules (8 solvent molecules and one LiPF_6_ unit) above the Li(100) slab, separated by a ∼15 Å vacuum region, constitutes an interfacial molecular layer. This approach is consistent with prior AIMD studies of SEI formation,^13,37–39^ where the focus is on electron-transfer-driven decomposition at the electrode surface rather than on reproducing bulk electrolyte properties such as molarity or density. All three compositions contain the same total number of molecules (8 solvent +1 salt), differing only in the identity of the additive species, so observed structural differences reflect additive chemistry rather than variations in molecular packing.

To focus on midtrajectory configurations after initial equilibration but before complete product formation, this work extracts 15 evenly spaced frames from the central 50% of each trajectory (2.25–4.5 ps), yielding 225 total configurations (75 per composition). Each configuration was encoded using SOAP descriptors as implemented in DScribe^40^ with a 5.0 Å cutoff radius, *n*_max_ = 8, and *l*_max_ = 6, generating rotation-invariant structural fingerprints that capture local coordination environments. The per-configuration SOAP vector was obtained by averaging over all atomic environments within each frame. SOAP vectors were standardized (zero mean, unit variance) and embedded into two-dimensional space using UMAP (*n*_neighbors_ = 10, min_dist_ = 0.1). Clustering was performed using HDBSCAN (min_cluster_size = 5). Bond statistics were computed using distance cutoffs validated against radial distribution functions: 1.7 Å (C–C), 1.6 Å (C–O, C–F), 2.4 Å (Li–O, Li–F), and 2.0 Å (P–F). Parameter sensitivity was assessed across UMAP (*n*_neighbors_ = 5–30, min_dist_ = 0.0–0.3) and HDBSCAN (min_cluster_size = 3–10) ranges (Figure S1).

To assess whether compositional separation reflects emergent structural divergence or inherent compositional encoding, three diagnostic analyses were performed. First, the initial configuration (*t* = 0) from each trajectory was encoded using the same SOAP parameters and standardized using the scaler fitted to midtrajectory data, then projected into the UMAP embedding via the trained transform. Second, to isolate emergent structural evolution from molecular identity encoding, the full SOAP-UMAP-HDBSCAN pipeline was repeated using SOAP descriptors centered exclusively on Li atoms. Since the Li slab is chemically identical across all three compositions, any separation in Li-centered SOAP space must reflect how different electrolyte molecules modify the Li coordination environment during decomposition, rather than encoding molecular identity. Third, time-resolved SOAP (timeSOAP) analysis^41^ was performed by computing the normalized angular distance between consecutive SOAP vectors for each sampled frame, yielding a scalar measure of structural fluctuation magnitude as a function of simulation time. Wasserstein distance analysis and bootstrap resampling stability are reported in the Supporting Information. The simulation models representing the Li(100)|electrolyte interface are shown in Figure 1.

**Figure 1:**
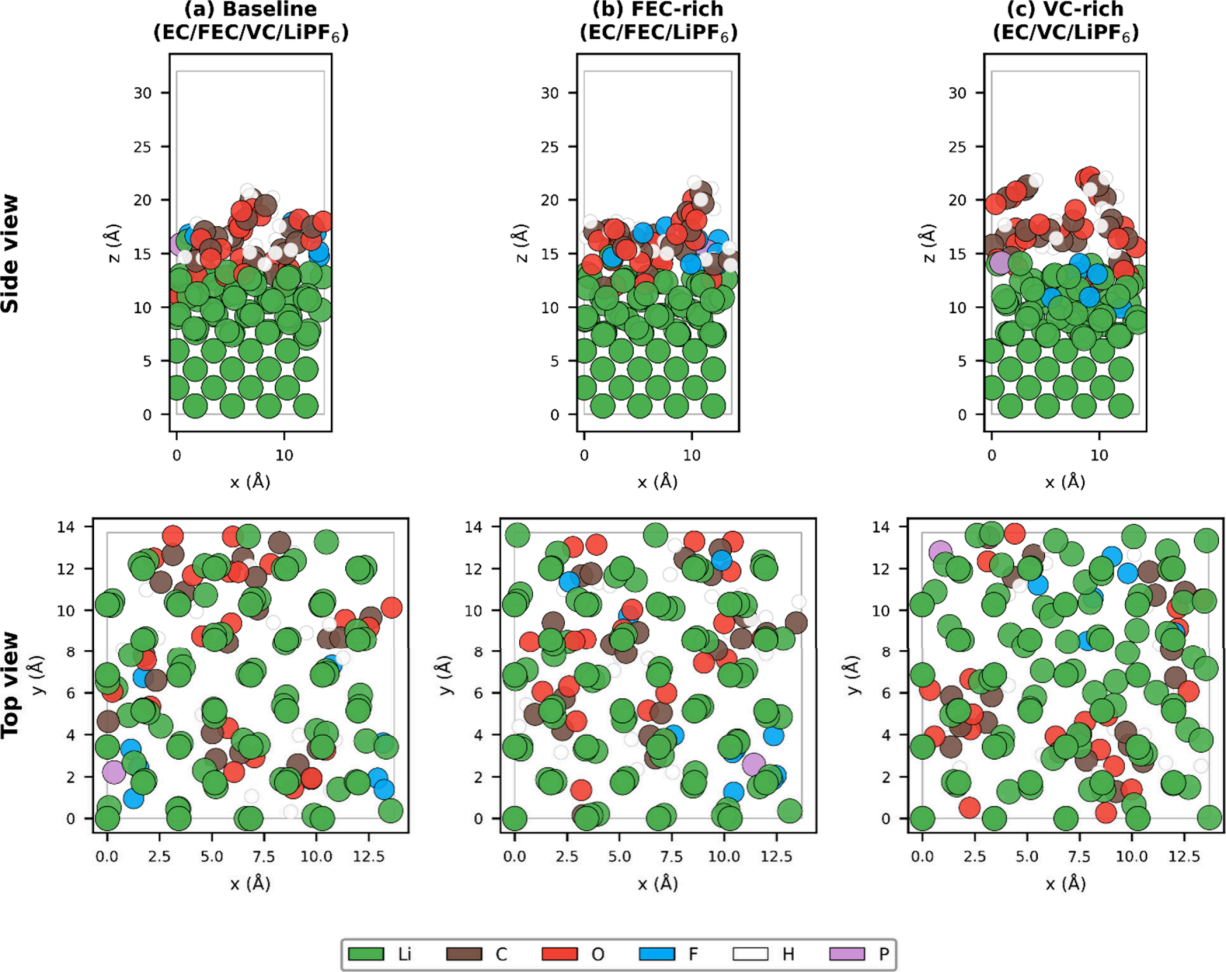
Simulation models of the Li(100)|electrolyte interface. (a–c) Representative snapshots of baseline (EC/FEC/VC/LiPF_6_), FEC-rich (EC/FEC/LiPF_6_), and VC-rich (EC/VC/LiPF_6_) compositions on a Li(100) slab. Li: green. C: brown. O: red. F: light blue. H: white. P: light purple. Bottom two Li layers are fixed; 15 Å vacuum separates periodic images.

The Li(100) surface was chosen because it represents a common facet of deposited lithium and provides a well-defined reference for comparing decomposition across compositions. Upon AIMD propagation, rapid electron transfer from the lithium surface to the electrolyte molecules was observed, initiating decomposition reactions consistent with previous computational studies.^37–39^ Rather than analyzing individual reaction events, this work focuses on characterizing the ensemble-level structural organization that emerges across independent trajectories. UMAP embedding of 225 midtrajectory configurations reveals complete separation of structural phase space by electrolyte composition (Figure 2). Baseline, FEC-rich, and VC-rich configurations occupy entirely nonoverlapping regions, with no mixing between composition classes. Within each composition, the five independent trajectories (15 frames each) form subclusters reflecting trajectory-specific structural evolution, revealing a hierarchical organization in which composition defines the accessible manifold while initial conditions determine the specific basin explored within that manifold. Notably, one baseline trajectory occupies an isolated region of UMAP space (upper right in Figure 2b), suggesting that specific initial configurations can access structural basins far from those explored by other trajectories of the same composition. This partitioning is robust to UMAP hyperparameter variation and HDBSCAN settings (Figure S1).

**Figure 2:**
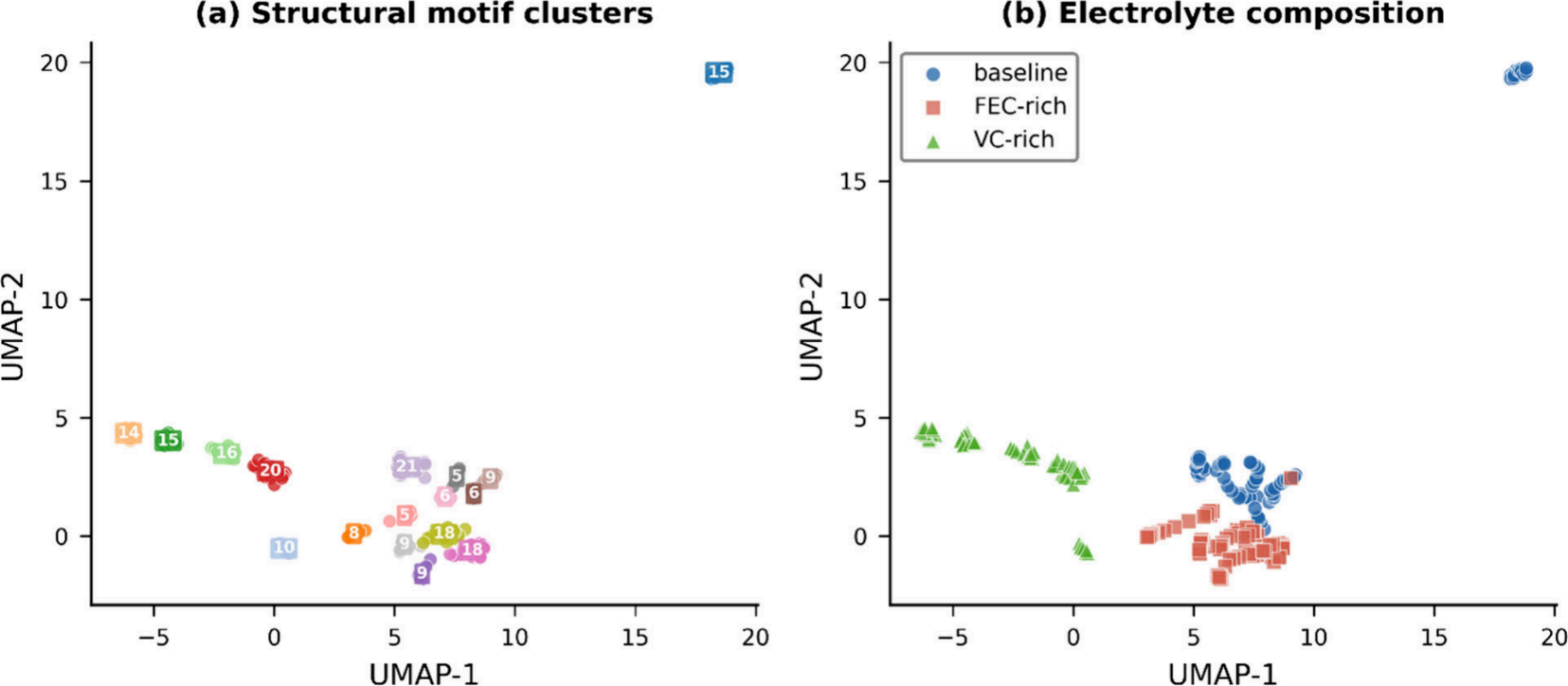
Compositional partitioning of structural phase space. (a) UMAP embedding of 225 midtrajectory AIMD configurations colored by HDBSCAN cluster assignment, revealing 17 discrete structural motifs with configuration counts annotated. (b) Same embedding colored by electrolyte composition, demonstrating complete separation of baseline (blue circles), FEC-rich (red squares), and VC-rich (green triangles) configurations.

Within each electrolyte formulation, HDBSCAN identifies 17 discrete structural motifs with strong composition dependence (Figure 3). The grouped bar chart (Figure 3a) reveals that most motifs are exclusively populated by a single composition, and the stacked percentage representation (Figure 3b) confirms that 15 of 17 motifs are 100% composition-exclusive, with only M13 (89% FEC-rich, 11% VC-rich) and M17 (94% FEC-rich, 6% baseline) showing any mixing. This near-total exclusivity reflects the distinct decomposition mechanisms of FEC and VC: FEC-exclusive motifs share characteristics consistent with carbonate-preserving S1 decomposition,^7^ while VC-exclusive motifs exhibit broader structural variability consistent with diverse oligomeric intermediates formed via polymerization-prone pathways. Baseline motifs access a hybrid structural subset, consistent with the presence of both FEC and VC in this formulation. The two motifs with minor mixing (M13, M17) suggest narrow regions of structural overlap where FEC-rich decomposition produces coordination environments partially accessible to VC-rich or baseline compositions.

**Figure 3:**
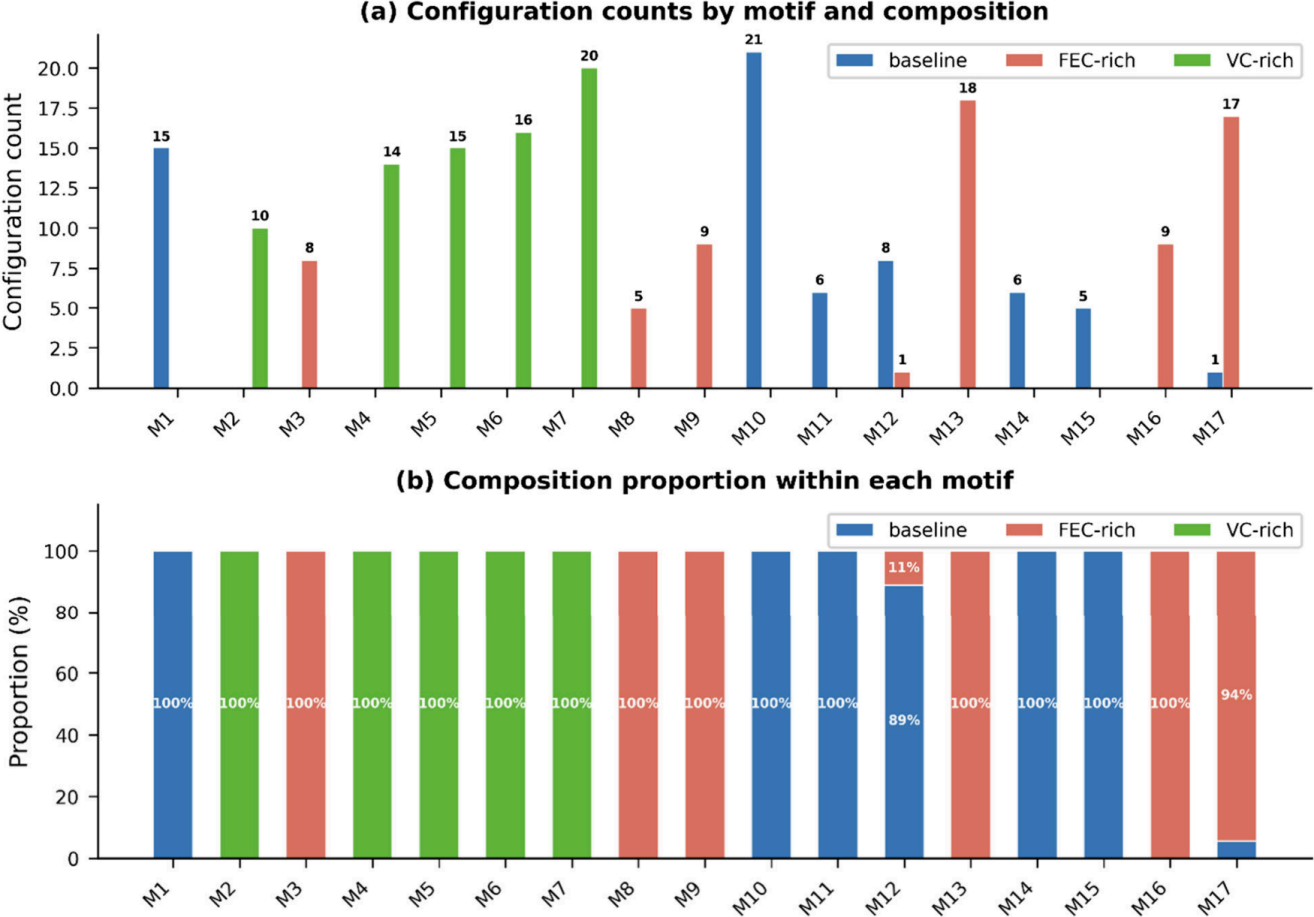
Composition-dependent motif populations. (a) Grouped bar chart showing configuration counts per structural motif (M1–M17) by electrolyte composition, with counts annotated. (b) Stacked percentage chart showing composition proportion within each motif. 15 of 17 motifs are composition-exclusive (100% from one electrolyte).

Low-dimensional embedding reveals clear separation between compositions (Figure 4), demonstrating that different electrolyte compositions define distinct regions of the underlying potential energy surface. This hierarchical structure (composition-level partitioning with trajectory-level basin organization) is interpreted through the lens of energy landscape concepts.^42,43^ Electrolyte chemistry controls which regions of the potential energy surface are kinetically accessible, while structural evolution within those regions remains confined on the 5 ps time scale, consistent with the concept of locally ergodic regions.^44^

**Figure 4:**
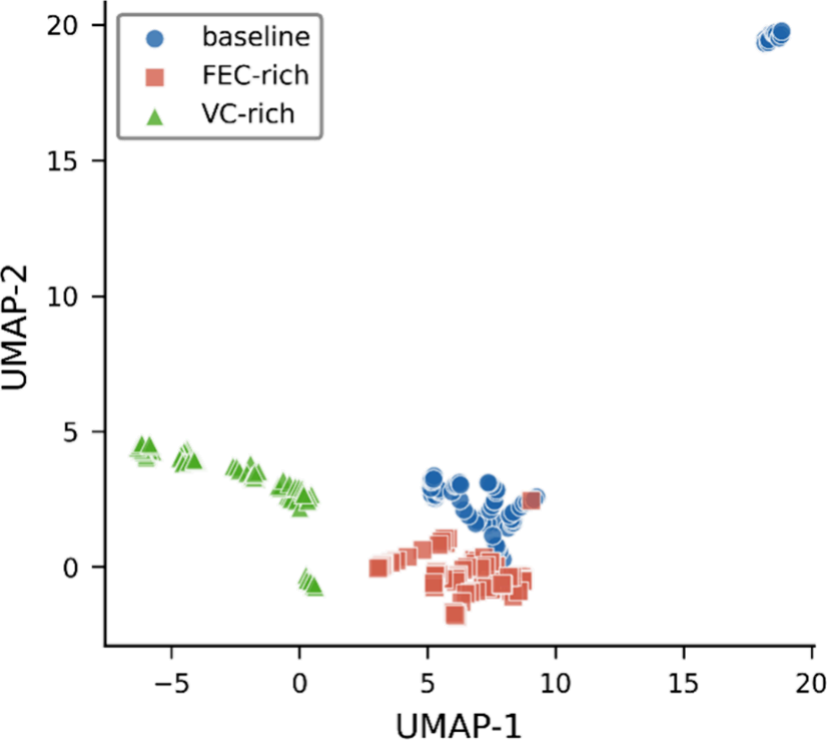
Composition-defined manifolds in configurational space. UMAP embedding of all sampled configurations colored by electrolyte composition (baseline, FEC-rich, and VC-rich).

Bond-level analysis reveals that composition-dependent phase space partitioning corresponds to systematic differences in local coordination chemistry (Figure 5). Li–O coordination emerges as the primary discriminating feature, with motif-averaged values ranging from approximately 8 to 44 bonds per configuration across the full motif set. Figure 5 organizes bond statistics by parent composition, enabling direct comparison: baseline motifs span an Li–O range of approximately 23–38, FEC-rich motifs range from 16 to 34, and VC-rich motifs exhibit the broadest variability (8–44), with motif M2 reaching the highest Li–O coordination observed in any composition. This broad VC-rich variability is consistent with VC’s known propensity for diverse coordination environments including both high-coordination polymeric species and low-coordination oligomeric intermediates.

**Figure 5:**
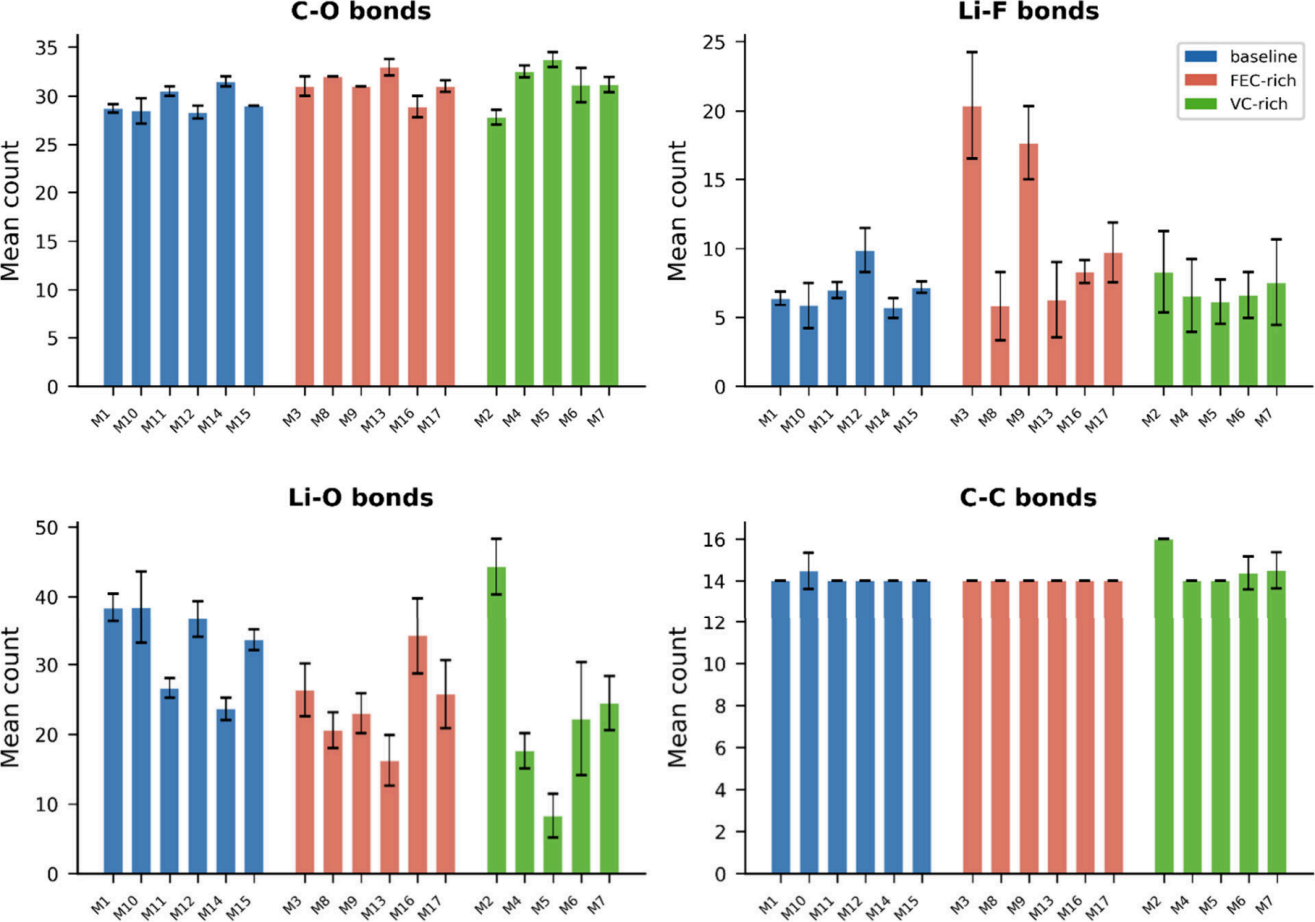
Motif-resolved bond statistics organized by composition. Mean counts (±s.d.) of C–O, Li–F, Li–O, and C–C bonds per motif, grouped by parent electrolyte composition (baseline, blue; FEC-rich, red; VC-rich, green). FEC-rich motif M3 shows dramatically elevated Li–F contacts (∼20), consistent with early stage LiF formation.

The Li–F panel reveals a striking composition-dependent signature: FEC-rich motif M3 exhibits approximately 20 Li–F contacts, far exceeding baseline (∼5–10) and VC-rich (∼6–9) motifs. This early stage Li–F coordination in FEC-rich systems is consistent with FEC’s known propensity for rapid C–F bond cleavage and LiF formation,^45–47^ and demonstrates that composition-dependent differences in fluorine chemistry are already detectable within the 5 ps simulation window. The electron stoichiometry for FEC reduction differs fundamentally from VC (three electrons per FEC versus one per VC^48^), which may contribute to the distinct structural organization observed for each composition. C–O bond counts are relatively constrained across compositions (∼28–34), and C–C bonds are remarkably uniform (∼14), indicating that the primary structural differentiation between compositions occurs through Li–O coordination variability and Li–F chemistry rather than through carbon backbone reorganization.

A critical question is whether the observed compositional separation reflects genuine structural evolution during decomposition or inherent compositional encoding in the SOAP descriptors. Figure 6 presents the diagnostic *t* = 0 projection analysis using all-atom SOAP descriptors. The initial configurations show partial compositional separation with substantially more overlap than midtrajectory configurations. VC-rich initial configurations already separate toward negative UMAP-1 values, consistent with different molecular species producing distinct SOAP fingerprints. However, baseline and FEC-rich initial configurations show more intermixing near the central UMAP region. At midtrajectory, all three compositions are more widely dispersed within their composition-specific regions and show cleaner separation. This indicates that some separation is inherent to compositional encoding in SOAP descriptors, while the increased dispersion and enhanced separation at midtrajectory provides evidence for additional emergent structural divergence during decomposition.

**Figure 6:**
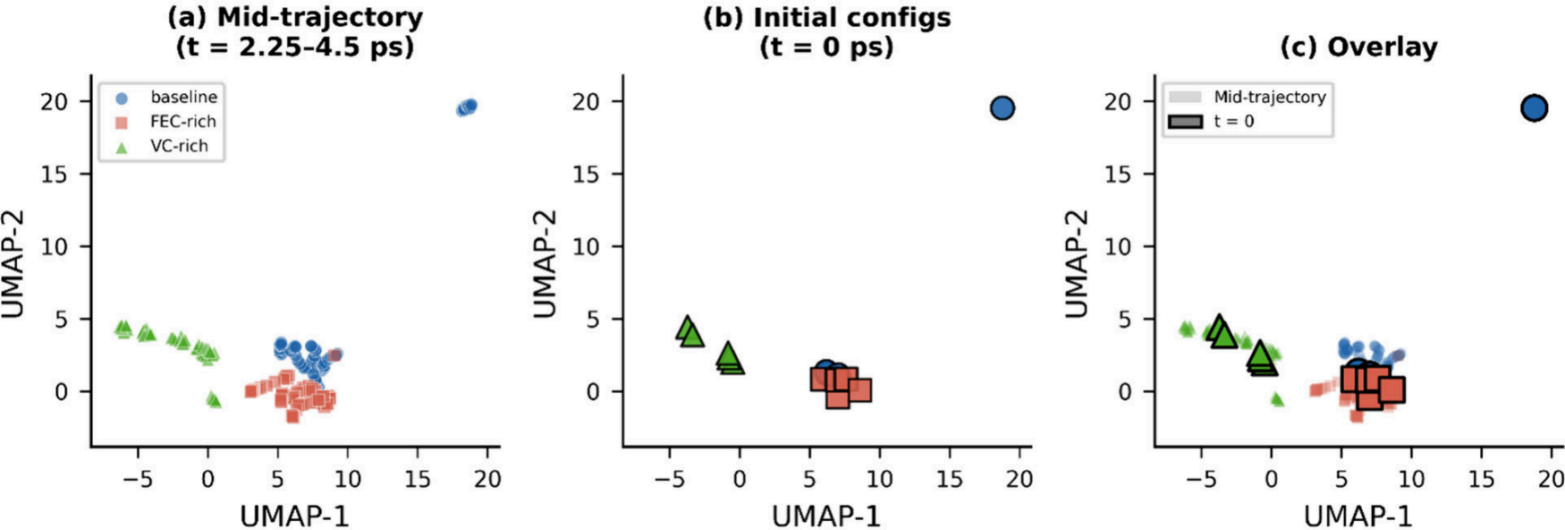
Diagnostic *t* = 0 projection analysis (all-atom SOAP). (a) Midtrajectory configurations in UMAP space. (b) Initial (*t* = 0) configurations projected into the same embedding. (c) Overlay showing that midtrajectory configurations are more dispersed and more cleanly separated than their initial counterparts.

To definitively isolate emergent structural evolution from compositional encoding, the analysis was repeated using SOAP descriptors centered exclusively on Li atoms (Figure S5). Since the Li slab is chemically identical across all three compositions, Li-centered SOAP cannot encode molecular identity. That is, any separation must reflect how different electrolytes physically modify the Li coordination environment. At *t* = 0, the Li-only SOAP projections for all 15 trajectories cluster together in a single compact region with extensive compositional intermixing, confirming that the Li surface initially appears structurally equivalent regardless of electrolyte composition. At midtrajectory, clear compositional separation emerges: VC-rich configurations migrate to distinct regions of Li-coordination space while baseline and FEC-rich occupy separate clusters. This result is consistent with different electrolyte molecules physically modifying the Li coordination environment in composition-specific ways during decomposition. While Li-centered SOAP descriptors can also encode the identity of neighboring molecular species within the cutoff radius, the absence of separation at *t* = 0 (when electrolyte molecules have not yet approached the surface), and its emergence at midtrajectory, combined with the composition-dependent bond formation observed in Figure 5, supports the interpretation that the separation reflects genuine chemical modification rather than passive molecular proximity. As a further test, the Li-only analysis was repeated with *r*_cut_ reduced from 5.0 to 2.5 Å, capturing only first-coordination-shell contacts (Figure S5). Compositional separation not only persists but strengthens (inter/intra ratio: 3.03 vs 2.61), while *t* = 0 intermixing remains complete (ratio 0.97), confirming that the observed divergence reflects composition-dependent bonding at the Li surface.

The time-resolved SOAP (τSOAP) analysis (Figure S3) provides a complementary picture of structural dynamics. All compositions show a burst of high structural fluctuation in the first ∼0.5 ps following electron transfer (τSOAP ∼ 0.008–0.012), reflecting rapid reorganization upon contact with the lithium surface. Baseline and FEC-rich exhibit higher peak τSOAP values than VC-rich during the first picosecond, indicating more dramatic initial structural rearrangement in fluorine-containing systems. Convergence to similar τSOAP values (∼0.002–0.003) by approximately 2 ps is consistent with the locally ergodic regime interpretation. That is, once the system has committed to a composition-specific structural basin, the rate of further structural change becomes composition-independent.

These results extend current mechanistic understanding in several ways. First, they provide ensemble-level statistical evidence that the distinct reaction channels identified for FEC versus EC/VC^7,8^ map onto distinct regions of structural configuration space. The S1/S2 pathway dichotomy corresponds to fundamentally different structural organization of the decomposing electrolyte, as reflected in the composition-exclusive motif assignments (15 of 17 motifs). Second, the compositional segregation in UMAP space, combined with the Li-only SOAP analysis showing that this separation emerges during decomposition from an initially equivalent Li surface, suggests that different reaction channels may be accessed from the earliest stages of decomposition. Third, the trajectory-level basin structure provides a framework for understanding SEI heterogeneity: different surface sites or local electrolyte configurations may access different basins within a composition-defined manifold, leading to spatially heterogeneous SEI even under nominally uniform conditions.

The connection between early time structural organization and long-time SEI architecture deserves consideration. Cryo-EM studies have established that FEC-containing electrolytes produce multilayer SEI with uniformly aligned crystalline grains, while EC-based electrolytes without FEC produce mosaic structures.^9–11^ Our observation that FEC-rich configurations cluster tightly in configuration space while VC-rich configurations span a broader structural range is consistent with this experimentally observed architectural divergence. Wagner-Henke et al.^23^ demonstrated that local Li^+^ concentration fundamentally affects whether layered or mosaic morphology emerges, indicating that early time coordination heterogeneity may propagate to macroscopic structure.

Several limitations merit acknowledgment. The 5 ps simulation time scale captures early structural organization but not complete SEI product formation, which occurs on nanosecond to microsecond scales.^20^ The interpretation that additives function as “pathway selectors” should be understood as preliminary evidence for composition-dependent structural organization during the earliest stages of interfacial reaction, rather than definitive determination of long-term decomposition outcomes. Importantly, the all-atom t = 0 projection analysis (Figure 6) reveals that some compositional separation is inherent to the SOAP encoding of different molecular species; however, the Li-only SOAP analysis (Figure S5) demonstrates that this initial bias is amplified by genuine, reaction-driven modification of the Li coordination environment during decomposition. The number of independent trajectories (5 per composition), while exceeding typical single-trajectory AIMD studies, may not exhaustively sample all accessible structural basins. Bootstrap resampling analysis, with a mean cluster purity of 0.959 across 100 iterations (Figure S4), demonstrates the stability of the main findings, but additional trajectories with more diverse initial configurations could reveal additional motifs. Moreover, the Li(100) surface represents one crystallographic facet; polycrystalline electrodes present multiple orientations that may access different structural manifolds.^49^

In summary, electrolyte composition appears to partition structural phase space during early interfacial reactions, with baseline, FEC-rich, and VC-rich electrolytes accessing largely distinct configuration manifolds characterized by different Li–O coordination environments and composition-dependent Li–F signatures. Elevated Li–F coordination in FEC-rich motifs within the 5 ps window suggests that fluorine chemistry is already active at the earliest stages of decomposition. Diagnostic analyses including *t* = 0 projection, Li-only SOAP, and time-resolved SOAP provide ensemble-level evidence for early stage structural differentiation that is consistent with pathway selection rather than simple descriptor-driven compositional bias. Collectively, these results suggest that electrolyte additives can influence which structural manifolds are accessed during the earliest interfacial reactions, and the ensemble AIMD plus SOAP–UMAP–HDBSCAN framework offers a useful approach for quantifying pathway diversity in reactive interfacial systems and informing electrolyte design.

## Supplementary Material



## Notes

UMAP parameter sensitivity analysis (Figure S1); Wasserstein distance evolution analysis (Figure S2); time-resolved SOAP (τSOAP) analysis of structural divergence dynamics (Figure S3); bootstrap resampling stability analysis (Figure S4); Li-only SOAP analysis isolating emergent structural evolution (Figure S5) (PDF)
