# Supplementary material for: Electrolyte Additives as Pathway Selectors in Early SEI Formation
Source: J Phys Chem Lett. 2026 May 7;17(20):5786–93. doi: 10.1021/acs.jpclett.6c00492 (PMC13200247; doi:10.1021/acs.jpclett.6c00492)
Supplement: Supplementary file 1 [file jz6c00492_si_001.pdf]

Supporting Information

Electrolyte Additives as Pathway Selectors in Early SEI Formation

Fernando A. Soto*

School of Science, Engineering, and Technology, Penn State Harrisburg, Middletown, Pennsylvania 17057, United States

*E-mail: fms5309@psu.edu

UMAP Parameter Sensitivity Analysis

To assess whether the compositional separation observed in Figure 2 of the main text is robust to the choice of UMAP hyperparameters, this work systematically varied $n_{neighbors}$ (5, 10, 15, 30) and min_{dist} (0.0, 0.1, 0.2, 0.3) and regenerated the UMAP embedding for each of the 16 parameter combinations (Figure S1). In all cases, the three electrolyte compositions remain clearly separated, confirming that the observed partitioning reflects genuine structural differences encoded in the SOAP descriptors rather than artifacts of a specific UMAP parameterization. At larger $n_{neighbors}$ values combined with larger min_{dist}, some overlap appears between baseline and FEC-rich configurations, which is scientifically expected given that the baseline composition contains both FEC and VC. VC-rich configurations remain distinctly separated across all parameter combinations.

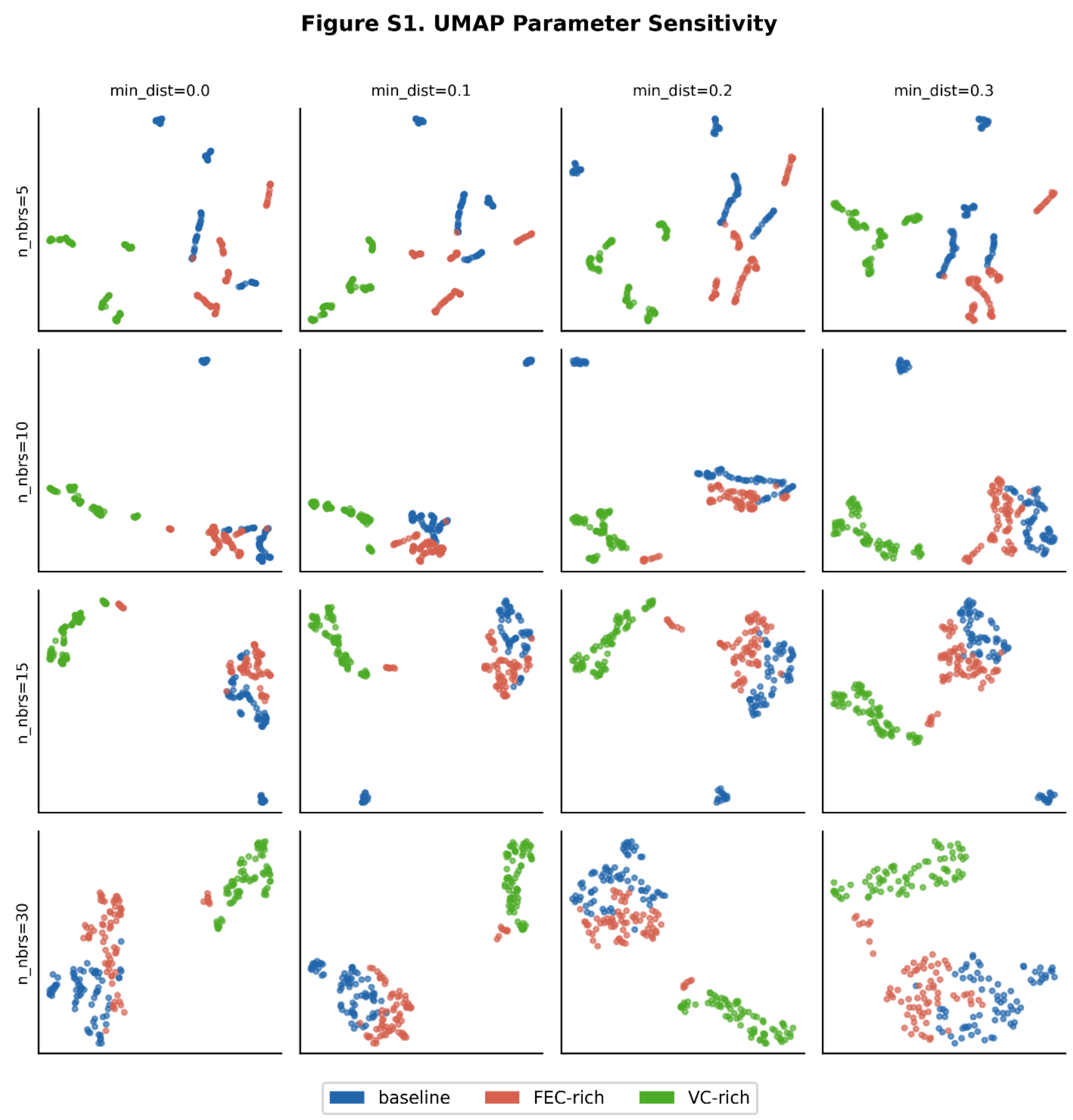

Figure S1. UMAP parameter sensitivity analysis. Each panel shows the UMAP embedding of 225 mid-trajectory configurations colored by electrolyte composition for a specific combination of $n_{neighbors}$ (rows) and min_{dist} (columns). Compositional separation is maintained across all 16 parameter combinations.

Wasserstein Distance Analysis

To provide a quantitative, UMAP-independent metric for structural separation between compositions, this work reports the Wasserstein (earth mover's) distance between pairwise composition SOAP distributions at t = 0 and at mid-trajectory (Figure S2). The per-dimension means are near zero for both conditions, with large standard deviations. This reflects the high dimensionality of the SOAP descriptor space: the vast majority of SOAP dimensions are uninformative for discriminating between compositions, while a small minority carry the compositional signal. The per-dimension averaging dilutes this signal, making the metric insensitive as a standalone measure. This work reports this analysis transparently as a complement to the UMAP-based, Li-only SOAP, and τSOAP-based analyses, which provide stronger evidence for composition-dependent structural evolution.

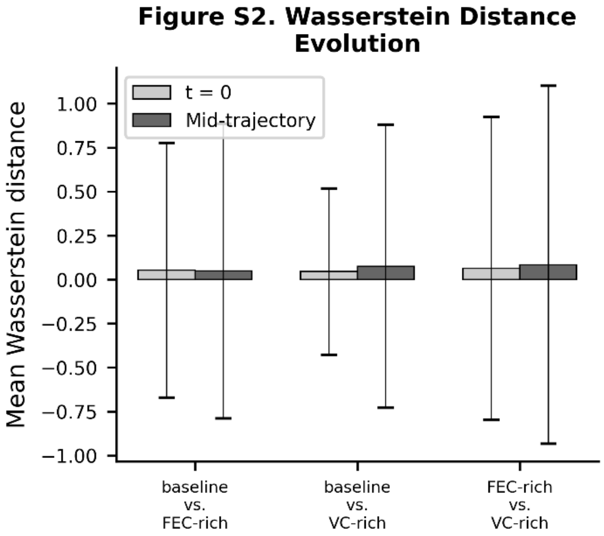

Figure S2. Wasserstein distance analysis. Mean per-dimension Wasserstein distance (± s.d.) between pairwise composition SOAP distributions at t = 0 (light gray) and mid-trajectory (dark gray).

Time-Resolved SOAP (τSOAP) Analysis

Time-resolved SOAP (τSOAP) analysis was performed to track continuous structural evolution across complete AIMD trajectories. The τSOAP descriptor, introduced by Caruso et al. *(1)*, computes the normalized angular distance between SOAP vectors at consecutive time frames: $d(\vec{a}, \vec{b}) = \sqrt{(2 - 2\, \vec{a} \cdot \vec{b} / \|\vec{a}\|\, \|\vec{b}\|)}$. This reduces the high-dimensional SOAP power spectrum to a single scalar per frame. SOAP parameters were set to $n_{max} = l_{max} = 4$ with $r_{cut} = 5.0$ Å, following the recommendation that reduced parameters yield consistent results at lower computational cost *(1)*. Each trajectory was downsampled to ~200 frames, and a Savitzky-Golay filter (window 11, polynomial order 2) was applied to reduce thermal noise.

Figure S3a shows individual trajectory τSOAP traces and composition-averaged traces. Figure S3b shows composition-averaged τSOAP with confidence bands. All compositions exhibit a burst of high structural fluctuation in the first ~0.5 ps (τSOAP ~ 0.008–0.012), reflecting rapid structural reorganization upon electron transfer from the lithium surface. Baseline and FEC-rich systems exhibit higher peak τSOAP values than VC-rich during the first picosecond, indicating more dramatic initial structural rearrangement in fluorine-containing systems. All compositions converge to similar τSOAP values (~0.002–0.003) by approximately 2 ps, consistent with the locally ergodic regime interpretation.

Figure S3. Time-Resolved SOAP (τSOAP) Analysis

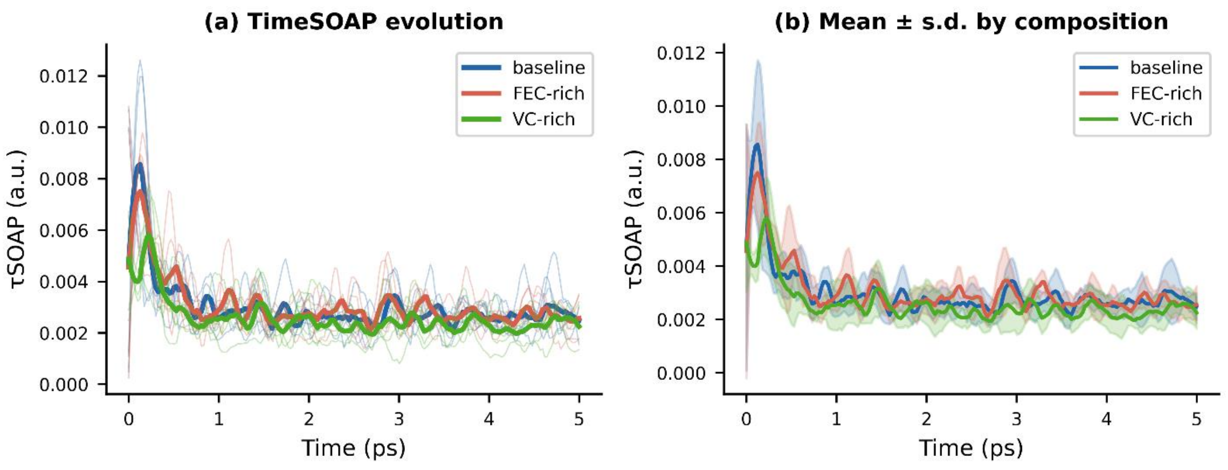

Figure S3. Time-resolved SOAP (τSOAP) analysis. (a) Individual trajectory traces (thin lines) and composition-averaged traces (thick lines). (b) Composition-averaged τSOAP with ± s.d. confidence bands.

Bootstrap Resampling Stability Analysis

Bootstrap resampling was performed to assess the robustness of compositional segregation. In each of 100 iterations, one trajectory was randomly removed from each composition (retaining 4 of 5), and the full UMAP-HDBSCAN pipeline was re-run. The mean cluster purity across all iterations is 0.959, indicating that 95.9% of configurations within each cluster belong to a single composition (Figure S4). The distribution is concentrated above 0.90, with even the lowest observed purity (~0.88) indicating robust segregation. This demonstrates that the compositional partitioning does not depend critically on any individual trajectory.

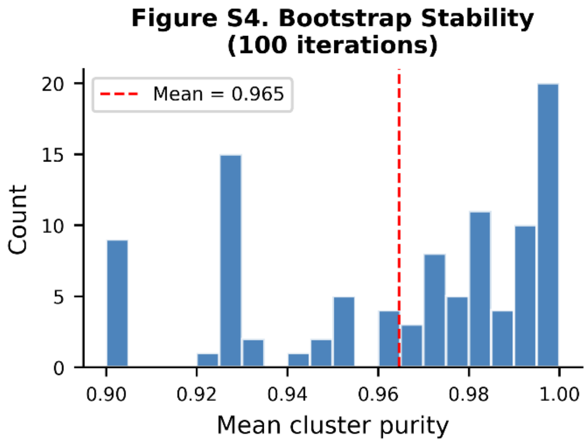

Figure S4. Bootstrap resampling stability analysis. Distribution of mean cluster purity across 100 iterations. Mean purity = 0.959 (red dashed line).

Li-Only SOAP Analysis: Isolating Emergent Structural Evolution

A central question is whether the compositional separation observed in the all-atom SOAP analysis (Figures 2 and 6) reflects emergent reaction-driven structural divergence or simply encodes the inherent molecular differences between FEC, VC, and EC in the SOAP descriptors. To definitively address this, the full SOAP-UMAP-HDBSCAN pipeline was repeated using SOAP descriptors centered exclusively on Li atoms.

The rationale is as follows: *the Li(100) slab is chemically identical across all three electrolyte compositions.* SOAP descriptors centered on Li atoms encode only the local coordination environment as seen from each Li center. That is, the arrangement of neighboring atoms (Li, C, O, H, F, P) within the cutoff radius. Because the Li centers themselves are identical across compositions, any separation in Li-centered SOAP space must arise from differences in how electrolyte molecules have arranged themselves around or reacted with the Li surface, rather than from encoding the identity of the electrolyte molecules themselves. For each frame, per-atom SOAP descriptors were computed for all atoms using the same parameters as the main analysis (r_{cut} = 5.0 Å, n_{max} = 8, l_{max} = 6). Only the rows corresponding to Li atom indices were retained, and these were averaged to produce a single Li-centered SOAP vector per frame. The same standardization, UMAP, and HDBSCAN pipeline was applied.

Figure S5 (panels a–c) presents the results at r_{cut} = 5.0 Å. Panel (a) shows the Li-only SOAP UMAP embedding for mid-trajectory configurations, which exhibits clear compositional separation: VC-rich configurations migrate to distinct regions while baseline and FEC-rich occupy separate clusters. Panel (b) shows the Li-only SOAP at t = 0: all 15 initial configurations cluster together in a single compact region with extensive compositional intermixing (inter/intra distance ratio = 1.03), confirming that the Li surface initially appears structurally equivalent regardless of electrolyte composition. At mid-trajectory, the separation ratio increases to 2.61 (a 2.54× increase). Panel (c) overlays both conditions.

While Li-centered SOAP descriptors eliminate molecular identity encoding, they can still capture neighboring molecular species within the cutoff radius that are merely proximate to the Li surface rather than chemically bonded. To distinguish bonding from passive proximity, the analysis was repeated with r_{cut} reduced from 5.0 Å to 2.5 Å (panels d–f), which captures only first-

coordination-shell contacts (Li–O bond distances ~2.0–2.4 Å; Li–F bond distances ~2.0–2.4 Å). At this reduced cutoff, t = 0 configurations remain fully intermixed (inter/intra ratio = 0.97, essentially unity), confirming that the Li first coordination shell is structurally identical across compositions before decomposition begins. At mid-trajectory, clear compositional separation emerges with a separation ratio of 3.03 (a 3.13× increase from t = 0, and stronger than the 5.0 Å result). The enhanced separation at the shorter cutoff indicates that the compositional signal is concentrated in the first coordination shell, where actual bonding occurs, rather than in outer shells where molecular proximity without bonding could contribute.

This result, combined with the composition-dependent bond formation observed in Figure 5 (particularly the ~20 Li–F contacts in FEC-rich motif M3), confirms that the observed phase space partitioning reflects genuine chemical modification of the Li surface. Different electrolyte compositions create fundamentally different bonding environments around initially equivalent Li atoms during the 5 ps decomposition window.

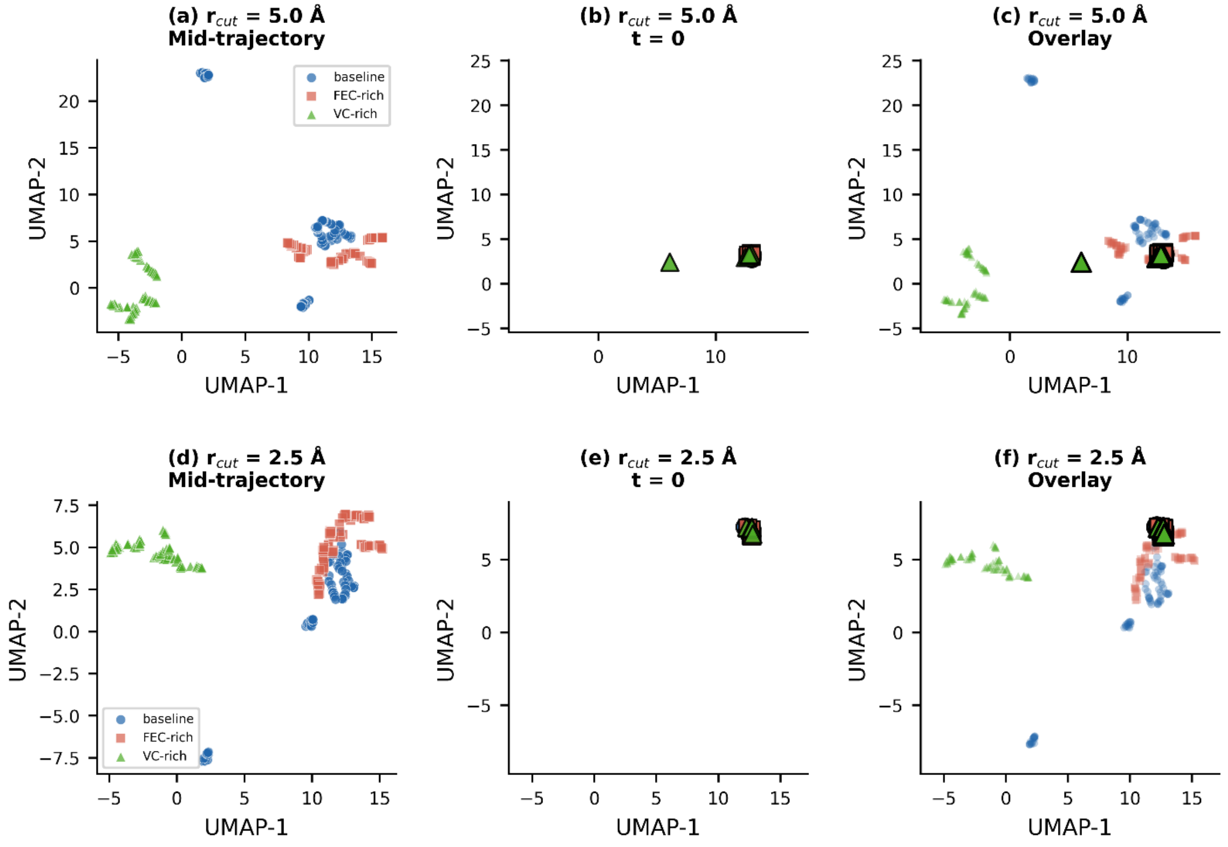

Figure S5. Li-only SOAP analysis comparing r_{cut} = 5.0 Å (top row) and r_{cut} = 2.5 Å (bottom row). (a, d) UMAP embedding of Li-centered SOAP vectors at mid-trajectory, colored by electrolyte composition (baseline: blue; FEC-rich: red; VC-rich: green), showing clear compositional separation at both cutoffs. (b, e) Li-centered SOAP at t = 0, showing all compositions clustered together with extensive intermixing (inter/intra distance ratio: 1.03 at 5.0 Å, 0.97 at 2.5 Å). (c, f) Overlay demonstrating that compositional separation in Li coordination space emerges during decomposition from an initially equivalent starting point. The separation ratio increases from t = 0 to mid-trajectory by 2.54× at r_{cut} = 5.0 Å and 3.13× at r_{cut} = 2.5 Å, confirming that the structural divergence is concentrated in first-coordination-shell bonding contacts rather than passive molecular proximity.

References

[1] Caruso, C.; Cardellini, A.; Crippa, M.; Rapetti, D.; Pavan, G. M. TimeSOAP: Tracking High-Dimensional Fluctuations in Complex Molecular Systems via Time Variations of SOAP Spectra. J. Chem. Phys. 2023, 158, 214302.
